# One-stop strategy combining pulmonary vein isolation with balloon-in-basket pulsed field ablation and left atrial appendage closure: a case report

**DOI:** 10.1093/ehjcr/ytaf627

**Published:** 2025-12-04

**Authors:** Lukas Urbanek, David Schaack, Melanie A Gunawardene, K R Julian Chun, Boris Schmidt

**Affiliations:** Cardioangiologisches Centrum Bethanien, Agaplesion Markus-Krankenhaus, Wilhelm-Epstein-Str. 4, Frankfurt/M 60431, Germany; Cardioangiologisches Centrum Bethanien, Agaplesion Markus-Krankenhaus, Wilhelm-Epstein-Str. 4, Frankfurt/M 60431, Germany; Cardioangiologisches Centrum Bethanien, Agaplesion Markus-Krankenhaus, Wilhelm-Epstein-Str. 4, Frankfurt/M 60431, Germany; Cardioangiologisches Centrum Bethanien, Agaplesion Markus-Krankenhaus, Wilhelm-Epstein-Str. 4, Frankfurt/M 60431, Germany; Klinik für Rhythmologie, Universitätsklinikum Schleswig-Holstein der Universität zu Lübeck, Ratzeburger Allee 160/Haus A, 23562 Lübeck, Germany; Cardioangiologisches Centrum Bethanien, Agaplesion Markus-Krankenhaus, Wilhelm-Epstein-Str. 4, Frankfurt/M 60431, Germany; Medizinische Klinik 3-Klinik für Kardiologie, Universitätsklinikum Frankfurt, Theodor-Stern-Kai 7, 60590 Frankfurt, Germany

**Keywords:** Balloon-in-basket pulsed field ablation, Pulmonary vein isolation, Left atrial appendage closure, Case report

## Abstract

**Background:**

Concomitant pulmonary vein isolation (PVI) and left atrial appendage closure (LAAC) are increasingly performed, with substantial experience from thermal ablation and limited early reports using pulsed field ablation (PFA). This is the first reported case of a combined procedure using a balloon-in-basket PFA system.

**Case summary:**

A 75-year-old man with symptomatic paroxysmal atrial fibrillation (AF) and recurrent macrohematuria under oral anticoagulation was referred for treatment. After a multidisciplinary team discussion, we opted for a one-stop approach combining PVI using a balloon-in-basket PFA system and LAAC. The procedure proceeded without complications, and at the end of the procedure and in follow-up imaging, no residual leak was detected.

**Discussion:**

This case highlights the feasibility of combining balloon-in-basket based PFA with LAAC in a single procedure. Performing ablation prior to LAAC may help avoid potential interactions between the ablation catheter and the occlusion device, while careful procedural planning can ensure optimal device sizing and complete sealing of the appendage, even in the context of post-ablation tissue changes.

Learning points

- One-stop strategy: Concomitant PVI and LAAC can be safely performed in a single session, reducing procedural burden.
- Novel ablation technique: Balloon-in-basket PFA represents a feasible alternative to thermal ablation for combined procedures.
- Procedural sequence matters: Performing PVI prior to LAAC may minimize the risk of catheter–device interaction and ensure reliable PVI.
- Device sizing: Pre-ablation measurement of LAA dimensions is essential to avoid device undersizing in the presence of post-ablation tissue edema.

## Introduction

Pulmonary vein isolation (PVI) and left atrial appendage closure (LAAC) are increasingly combined in a single procedure to provide rhythm control and stroke prevention while limiting exposure to oral anticoagulation. Most available data derive from thermal ablation techniques,^1,2^ which may alter left atrial appendage (LAA) dimensions and impact device sizing.^3^ Pulsed field ablation (PFA) has emerged as a non-thermal energy source with favorable safety and efficacy profiles, but evidence on its integration with concomitant LAAC remains scarce. We present the first reported case of a combined one-stop strategy using a balloon-in-basket PFA system and LAAC, highlighting procedural feasibility, technical considerations, and potential clinical implications.

## Summary figure

**Figure:**
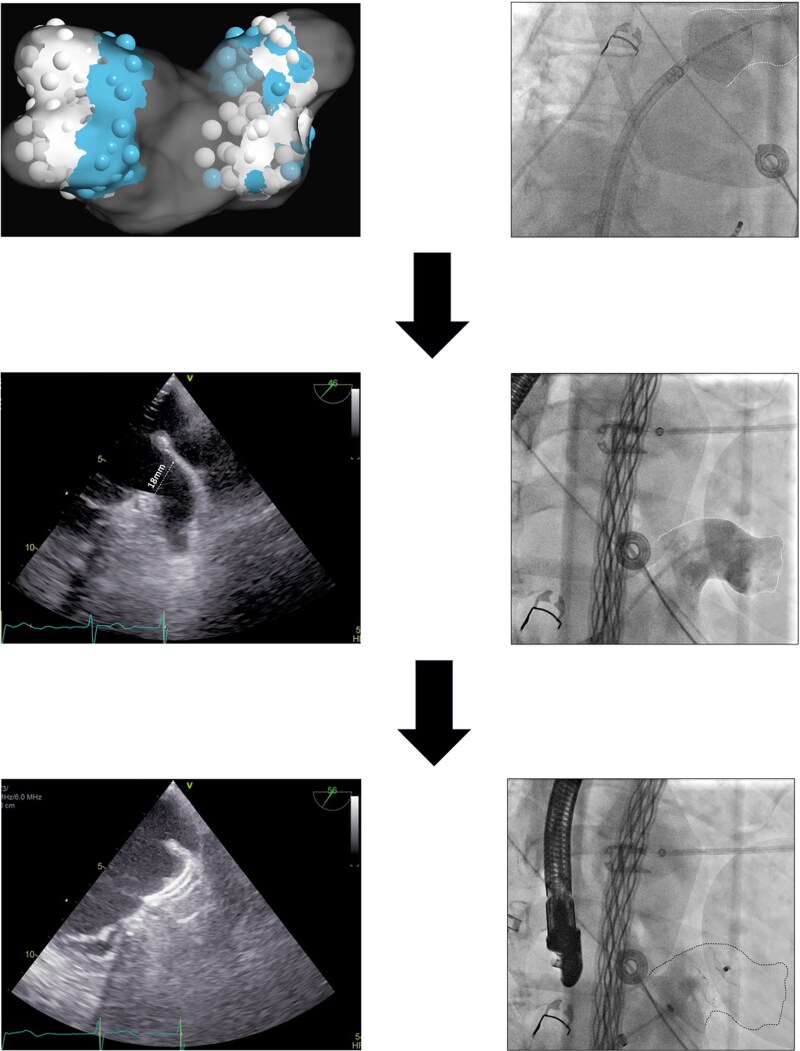


## Case presentation

**Figure 1:**
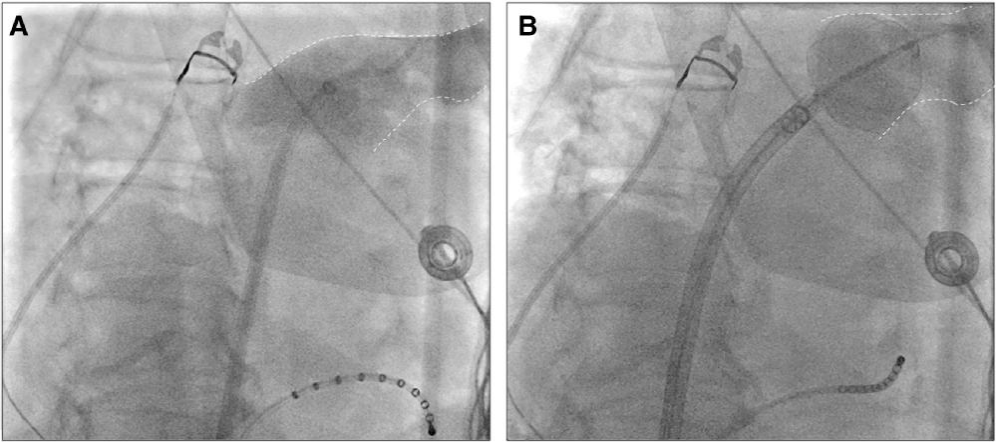
(*A*) Selective pulmonary vein angiography of the left superior pulmonary vein in 30° right anterior oblique projection. (*B*) Occlusion angiography using the balloon-based PFA catheter, demonstrating adequate vein occlusion in the same 30° RAO projection. PFA, pulsed field ablation; RAO, right anterior oblique.

**Figure 2:**
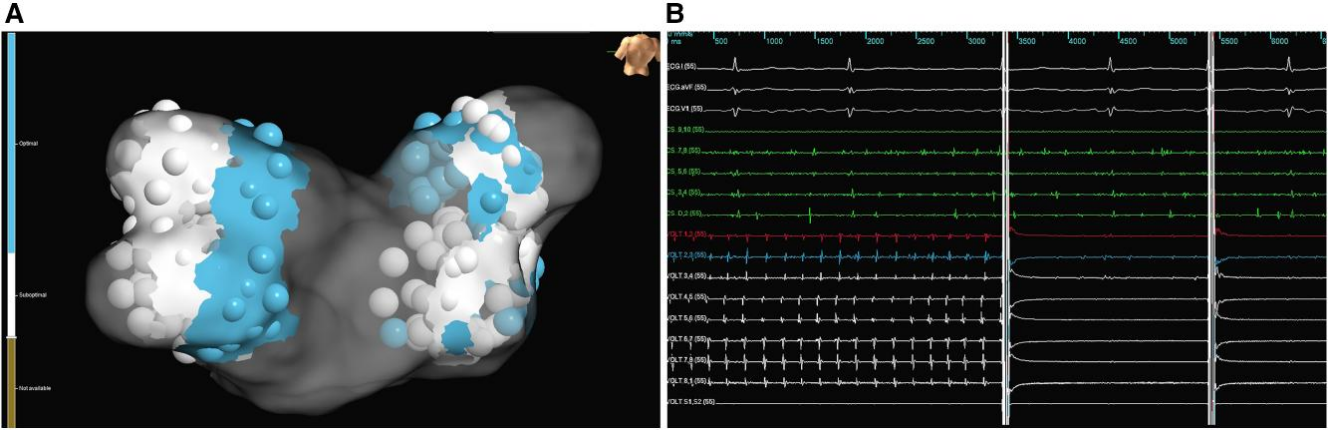
(*A*) Electroanatomical map of the left atrium showing ablation points. Blue dots indicate areas of higher tissue contact, while white dots represent sites with lower contact. Additional lesions with slight balloon protrusion into the pulmonary vein are also marked in white. (*B*) Real-time pulmonary vein isolation during the first pulse of the initial application.

We report the case of a 75-year-old man with paroxysmal AF for 7 years, with progressively increasing frequency and duration, and an early recurrence following recent electrical cardioversion. He had an elevated thromboembolic risk (CHA₂DS₂-VA-Score 3) and recurrent macrohematuria under oral anticoagulation, which had led to multiple postponements of planned PVI. Physical examination and baseline blood work were unremarkable. Preprocedural transesophageal echocardiography (TEE) ruled out intracardiac thrombus and the LAA ostium and the landing zone were measured. No additional preprocedural imaging was performed and baseline ECG showed sinus rhythm. After multidisciplinary discussion and given the combined need for rhythm control and stroke prevention while avoiding long-term anticoagulation, a combined strategy of PVI using a novel PFA balloon-in-basket catheter (VOLT™, Abbott) and left atrial appendage closure (LAAC) with an Amulet™ (Abbott) device was pursued aiming to minimize procedural burden and antithrombotic exposure. Under conscious sedation with propofol and midazolam, single transseptal access was obtained and selective PV angiographies (*Figure 1A*) were performed. After occlusion angiography (*Figure 1B*) all four pulmonary veins were successfully isolated (real time isolation *Figure 2B*) using the VOLT™ PFA balloon catheter (Abbott). In total, 6 applications were delivered per vein: 4 at an antral position (*Figure 2A*; blue dots), and, after slight deflation of the balloon, two additional applications at a more ostial level with only a slight protrusion of the balloon into the pulmonary vein (*Figure 1A*; white dots), which we call an olive configuration.

**Figure 3:**
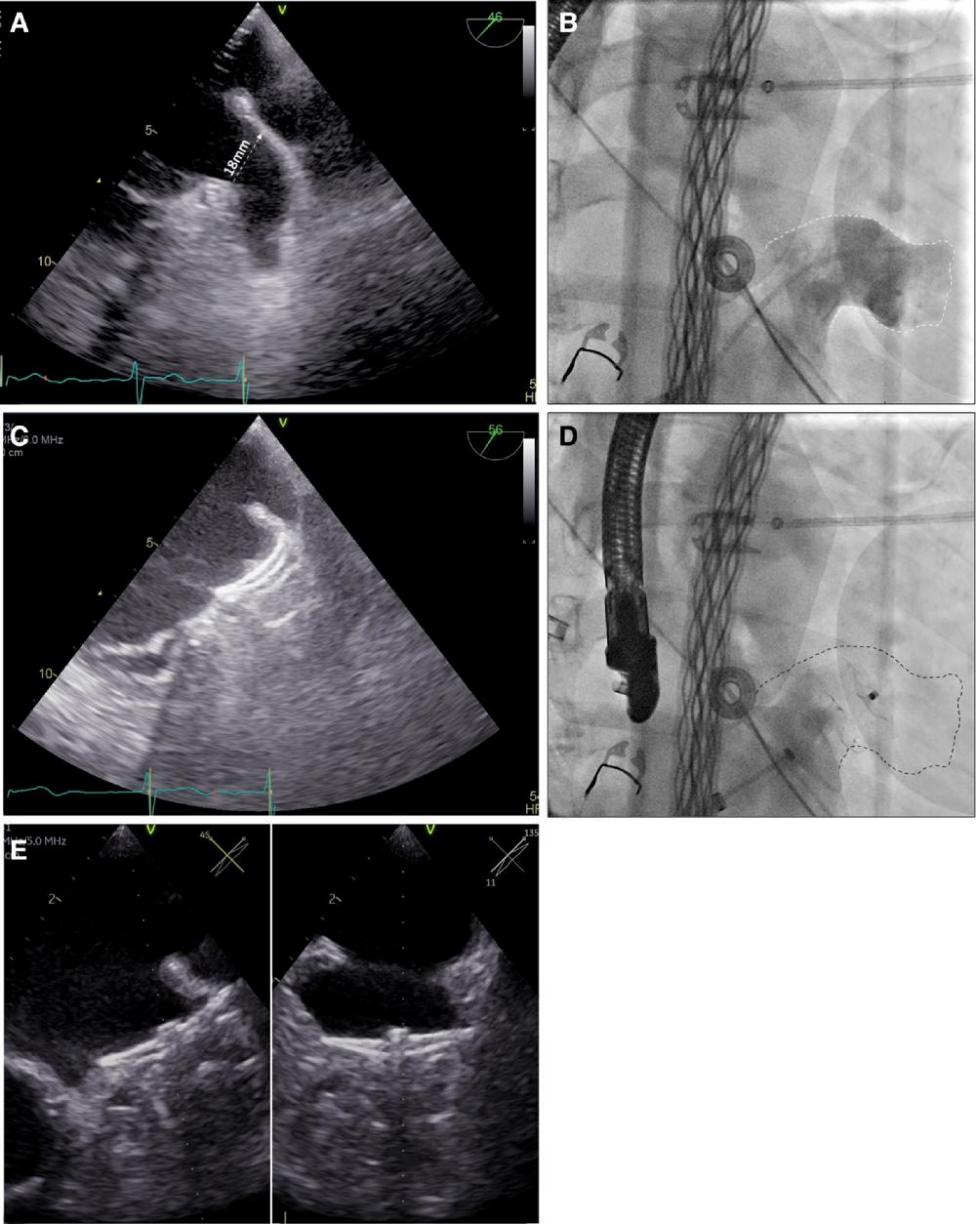
(*A*) TEE showing the LAA after ablation with a landing zone diameter of 18 mm (white arrow). (*B*) Fluoroscopic image in RAO 30°/cranial 15° projection with contrast angiography confirming a landing zone of 18 mm. (*C*) TEE demonstrating complete sealing of the LAA and a device compression of 21 mm. (*D*) Fluoroscopic RAO 30°/cranial 15° view with angiography confirming complete LAA occlusion with no residual contrast flow into the appendage. (*E*) Follow-up imaging after 6 weeks showing complete sealing of the LAA without residual leakage. LAA, left atrial appendage; RAO, right anterior oblique; TEE, transesophageal echocardiography.

Subsequently, left atrial appendage (LAA) angiography demonstrated a landing zone diameter of 18 mm (*Figure 3B*), which was consistent with transesophageal echocardiographic (TEE) measurements (*Figure 3A*). Comparative TEE assessment of the LAA before and after ablation revealed only a mild ridge swelling without any change in LAA dimensions. A 25 mm Amulet™ device (Abbott) was successfully deployed under TEE and fluoroscopic guidance. Final imaging confirmed correct positioning with a device compression of 20% (*Figure 3C*), no significant peri-device leak in TEE (visually and with color Doppler) and in LAA angiography (*Figure 3D*) and a successful tug test was performed. The in-hospital course was uneventful. The patient was discharged on apixaban, which was continued for 3 months and subsequently transitioned to single antiplatelet therapy with aspirin. At the 6-week follow-up, TEE confirmed stable device position without any peri-device leak (*Figure 3E*).

## Discussion

This case highlights the feasibility of a one-stop approach combining PFA and LAAC in patients with paroxysmal AF and contraindications to long-term anticoagulation. The novel balloon-in-basket PFA catheter offers a promising alternative to thermal ablation, with potentially favorable safety and efficacy profiles.

Previous studies have reported a higher incidence of peri-device leaks (PDL) on follow-up imaging in patients undergoing concomitant thermal PVI followed by LAAC.^3,4^

This phenomenon is most likely related to post-ablation tissue edema, which may result in underestimation of the true LAA diameter and subsequent device undersizing.^3^ Supporting this hypothesis, a randomized trial comparing an ‘thermal ablation-first’ vs. a ‘LAAC-first’ approach demonstrated significantly higher PDL rates and lower device compression ratios in the ablation-first group.^5^ Whether such edema formation also occurs following non-thermal PFA remains to be fully elucidated; however, early case reports^6^ and initial studies^7^ have described evidence of tissue edema even with PFA. In our case, we opted for an ablation-first strategy for three primary reasons. First, reports have described potential short-circuiting of PFA energy during ablation of the left superior pulmonary vein (LSPV) with the pentaspline PFA catheter (Farawave™, Boston Scientific) in patients with a pre-existing LAAC device.^8,9^ This appears to be particularly relevant with lobe-and-disk occluders, where protrusion of the disk into the left atrial cavity may bring it into contact with the PFA catheter’s splines during energy delivery when ablating the LSPV.^8^ Although it remains unclear whether similar interactions could occur with the balloon-in-basket PFA system, we performed PVI prior to LAAC to eliminate any potential risk of interference between the PFA balloon and the occlusion device.

Second, based on our experience with the Farawave™ PFA catheter, even in the presence of mild ridge edema, the dimensions of the LAA—particularly the landing zone—remain unchanged. This was also the case in the present case. Consistently, follow-up TEE at 6 weeks confirmed stable device position without any peri-device leak, supporting the accuracy of the initial LAA measurements despite preceding PFA.

Third, we performed pre-ablation measurements of the LAA dimensions to ensure accurate device sizing and to avoid undersizing, even in the presence of relevant tissue edema.

While the one-stop strategy offers the advantage of combining rhythm control and stroke prevention within a single session, it also involves specific procedural trade-offs compared with a staged approach. Performing both procedures during one sitting reduces the need for repeated vascular access, sedation, and hospitalization, thereby minimizing cumulative procedural burden and antithrombotic exposure—an aspect particularly relevant in patients with bleeding complications under oral anticoagulation, as in our case.

However, a staged approach with ablation performed first may allow reassessment of the LAA after resolution of post-ablation edema, potentially improving the accuracy of device sizing and reducing the risk of undersizing or peri-device leak. The main drawback of this strategy is the need for continued oral anticoagulation during the interval between procedures. Conversely, performing LAAC first could expose the implanted device to potential electrical or mechanical interaction during subsequent ablation. Moreover, after the first procedure, transseptal re-access may be technically more challenging during the second intervention.

Thus, both strategies have distinct advantages and limitations that should be individualized based on patient profile and bleeding risk, and further studies are needed to define the optimal sequencing and imaging timing in combined PFA–LAAC procedures.

Beyond procedural and clinical considerations, the generalizability of this combined approach may also depend on health system–specific reimbursement structures. In some settings, single combined procedures may not yet be uniformly reimbursed or may vary in coverage depending on inpatient vs. day-case performance and device-specific policies. These differences could influence procedural adoption and should be considered when interpreting feasibility and cost-effectiveness across healthcare systems.

## Conclusion

This case illustrates the feasibility and safety of a one-stop approach combining PFA and LAAC using a balloon-in-basket PFA system. Performing PVI prior to LAAC may reduce the risk of interaction between the ablation catheter and the occluder device during ablation, while careful procedural planning ensures accurate sizing and effective occlusion. Pre-ablation measurement of the LAA diameter is essential to ensure proper sizing and to avoid undersizing of the occluder in the presence of tissue edema. In selected patients with contraindications to long-term anticoagulation, this combined strategy may represent a feasible and potentially valuable therapeutic option for both rhythm control and stroke prevention, while reducing overall procedural burden. However, larger studies are warranted to confirm its safety and efficacy.

## Supplementary Material

ytaf627_Supplementary_Data

## Data Availability

The data underlying this article are available from the corresponding author upon reasonable request.

## Lead author biography

Lukas Urbanek is a cardiologist and researcher at Cardiologisches Centrum Bethanien (CCB) in Frankfurt, Germany. His clinical and scientific focus lies on catheter ablation of atrial fibrillation and left atrial appendage closure.

## Notes

**Consent:** Informed consent was obtained from the patient in accordance with COPE guidelines.

**Funding:** None declared.
