# Supplementary material for: One-stop strategy combining pulmonary vein isolation with balloon-in-basket pulsed field ablation and left atrial appendage closure: a case report
Source: Eur Heart J Case Rep. 2025 Dec 4;9(12):ytaf627. doi: 10.1093/ehjcr/ytaf627 (PMC12715504; doi:10.1093/ehjcr/ytaf627)
Supplement: ytaf627_Supplementary_Data [file ytaf627_supplementary_data.docx]

### Timeline

- **08/2017** – Infection-triggered atrial fibrillation due to pneumonia; initiation of oral anticoagulation with apixaban; spontaneous conversion to sinus rhythm.
- **10/2017** – Coronary angiography with exclusion of coronary artery disease
- **2017–2024** – Symptom-free interval.
- **11/2024** – Recurrence of AF; electrical cardioversion to sinus rhythm.
- **02/2025** – AF recurrence; catheter ablation scheduled.
- **04/2025** – Multiple postponements of ablation due to recurrent macrohematuria; decision for a concomitant procedure (AF ablation + left atrial appendage closure).
- **06/2025** – Successful concomitant pulsed field ablation–based pulmonary vein isolation and left atrial appendage closure performed.
